# A Green Ultrasound-Assisted Extraction Optimization of the Natural Antioxidant and Anti-Aging Flavonolignans from Milk Thistle *Silybum marianum* (L.) Gaertn. Fruits for Cosmetic Applications

**DOI:** 10.3390/antiox8080304

**Published:** 2019-08-14

**Authors:** Samantha Drouet, Emilie A. Leclerc, Laurine Garros, Duangjai Tungmunnithum, Atul Kabra, Bilal Haider Abbasi, Éric Lainé, Christophe Hano

**Affiliations:** 1 Laboratoire de Biologie des Ligneux et des Grandes Cultures (LBLGC), INRA USC1328, Université d’Orléans, Pôle Universitaire d’Eure et Loir, 21 rue de Loigny la Bataille, F-28000 Chartres, France; 2 Bioactifs et Cosmétiques, Centre National de la Recherche Scientifique (CNRS) – Groupement de Recherche 3711, Université d’Orléans, 45067 Orléans Cedex 2, France; 3 Department of Pharmaceutical Botany, Faculty of Pharmacy, Mahidol University, 447 Sri-Ayuthaya Road, Rajathevi, Bangkok 10400, Thailand; 4 Inder Kumar Gujral Punjab Technical University, Kapurthala, Punjab 144603, India; 5 Kota College of Pharmacy, Kota Rajasthan 325003, India; 6 Department of Biotechnology, Quaid-i-Azam University, Islamabad 45320, Pakistan

**Keywords:** *Silybum marianum*, Silymarin, flavonolignans, ultrasound-assisted extraction, design of experiment, antioxidant, anti-aging

## Abstract

*Silybum marianum* (L.) Gaertn. (aka milk thistle) constitutes the source of silymarin (SILM), a mixture of different flavonolignans and represents a unique model for their extraction. Here we report on the development and validation of an ultrasound-assisted extraction (UAE) method of *S. marianum* flavonolignans follow by their quantification using LC system. The optimal conditions of this UAE method were: aqueous EtOH 54.5% (v/v) as extraction solvent, with application of an ultrasound (US) frequency of 36.6 kHz during 60 min at 45 °C with a liquid to solid ratio of 25:1 mL/g dry weight (DW). Following its optimization using a full factorial design, the extraction method was validated according to international standards of the association of analytical communities (AOAC) to ensure precision and accuracy in the quantitation of each component of the SILM mixture. The efficiency of this UAE was compared with maceration protocol. Here, the optimized and validated conditions of the UAE allowed the highest extraction yields of SILM and its constituents in comparison to maceration. During UAE, the antioxidant capacity of the extracts was retained, as confirmed by the in vitro assays CUPRAC (cupric ion reducing antioxidant capacity) and inhibition of AGEs (advanced glycation end products). The skin anti-aging potential of the extract obtained by UAE was also confirmed by the strong in vitro cell-free inhibition capacity of both collagenase and elastase. To summarize, the UAE procedure presented here is a green and efficient method for the extraction and quantification of SILM and its constituents from the fruits of *S. marianum*, making it possible to generate extracts with attractive antioxidant and anti-aging activities for future cosmetic applications.

## 1. Introduction

**Figure 1:**
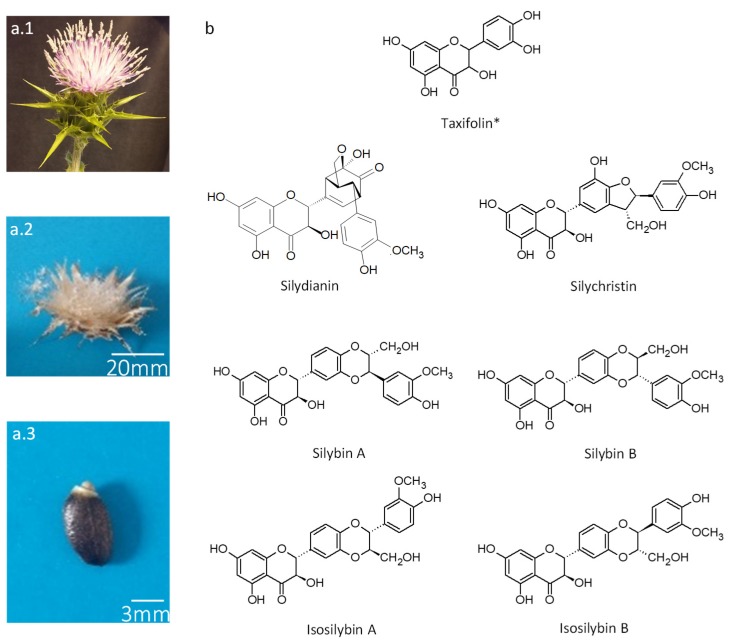
(**a**) Representative pictures of a flowering capitulum (**a.1**), of an open mature capitulum bearing mature fruit (achenes) (**a.2**); and of a mature fruit (achene) (**a.3**) of milk thistle (*S. marianum*) (**b**) Structures of the six flavonolignans (silychristin (SILC), silydianin (SILD), silybin A (SILA), silybin B (SILB), isosilybin A (ISILA) and isosilybin B (ISILB)) and one flavonoid (*, taxifolin, TAX)) from the silymarin (SILM) mixture extracted from milk thistle (*S. marianum*) mature fruit (achene).

*Silybum marianum* (L.) Gaertn. aka Milk thistle (Asteraceae family) is one of the oldest herbal medicines, growing as an annual, winter annual or biennial herb, depending on climate [1]. Its fruit accumulates high levels of silymarin (SILM), a complex mixture of taxifolin-derived flavonolignans comprising silydianin (SILD), silychristin (SILC), silybins A (SILA) and B (SILB) and isosilybins A (ISILA) and B (ISILB) (Figure 1) [2,3]. These compounds are the result of oxidative coupling between a flavonoid part (i.e., taxifolin) and a lignan precursor (i.e., coniferyl alcohol) [4]. *S. marianum* constitutes a unique source of these flavonolignan mixture deriving from taxifolin [5], and is thus considered an attractive raw resource to extract and valorise these compounds for industrial applications.

Milk thistle is one of oldest medicinal plants, has been used for centuries to cure many diseases, and has traditionally been used in the European Pharmacopoeia as a liver detoxifier [6,7,8], as well as a unique remedy against *Amanita phalloides* intoxication [9,10]. For many decades, milk thistle was cultivated as a crop and used to cure of hepatobiliary diseases [11]. Some established commercial cultivars are available in Europe for this purpose. However, other biological activities have been ascribed to SILM. SILM has been investigated for numerous pharmacological actions that could be of benefit to human health, for example in the prevention of prevent ovarian [12] or breast [13] cancers.

More recently, SILM has attracted growing interest due to its effective antioxidant and anti-aging activities [14,15,16,17,18,19,20]. In particular, silybins, described as the most active constituents of SILM, display a wide range of biological activities, including antioxidant and skin anti-inflammatory properties [21]. In cosmetics, the anti-aging activities of plant extracts are linked to their capacity to decrease skin damage caused by reactive oxygen and/nitrogen species (ROS/RNS), along with their aptitude for controlling the activity of various enzymes involved in skin aging progression; for example, due to their capacity to inhibit elastase or collagenase involved in the cleavage of extracellular matrix components. For instance, Vostalova et al. [16] reported on the inhibitory actions of SILM, its flavonolignans and some related derivatives toward the collagenase and elastase and evidenced diverse affinities against these enzymes. SILM also confers UV-B protection [22] and has been proposed to protect skin against sunburns or skin cancers [23]. All these biological activities are of special interest for cosmetic applications and trigger the necessity of developing efficient green extraction protocols of SILM.

Many green extraction methods of plant natural products using, for example, microwave-assisted extraction (UAE) [24,25], pressured liquid extraction [26], cellulase-assisted [27] or ultrasound-assisted [28] extractions have already been published. In the present study, we focused on UAE, which is one of the most simple and economical methods for improving the extraction yield of plants [29]. It generally presents a shorter extraction time with a reduced use of solvent as compared to other conventional extraction methods, making it a green extraction procedure that can be rapidly upscaled for industrial purposes [30]. These effects of US are explained by improved mass transfer and cell disruption, better cell penetration of the solvent improving the extraction, and also by capillary effects, limiting the degradation of the constituents [31].

To obtain the optimal conditions for extraction yields, a design of experiment (DOE) coupled with response surface methodology (RSM) was used to determine and optimize the values of independent parameters, such as extraction time, aqueous ethanol (aqEtOH) concentration and US frequency influencing the SILM extraction. Bioassays were performed to evaluate the evolution of antioxidant and anti-aging activities of the extracts as a function of the extraction conditions. Correlations linking phytochemical profile and biological activities of the extracts were calculated. The optimized extraction protocol was also used to investigate the SILM content and composition of 4 commercial milk thistle cultivars.

## 2. Materials and Methods

### 2.1. Plant Material

All milk thistle (*S. marianum* (L.) Gaertn.) fruits were derived from commercial cultivars provided by PMA28 (Varize, France). All the cultivars were grown from seed under the same growing conditions of an experimental site located in Chateaudun (48°04′18″ N/1°20′19″ E/127 m, Eure-et-Loir, Centre-Val de Loire, Chateaudun, France). Sowings, 350 seeds per m^2^, were performed on the 30th of March. Fertilization was realized immediately after sowing with nitrogen 30, potassium 20 and phosphorus 20 units per hectare. The soil was of clay loam type with a granulometry of ca. 25% 2000–63 µm, 50% 63–2 µm, and 25% <2 µm particles, and a pH of around 7.0. The final harvest took place on the 15th of August. No visible disease nor insect attack were detected. Over the cultivation period site received 225.5 mm of rainfall and the average day temperature was 15.7 °C.

### 2.2. Chemicals

All solvents and reagents for extraction and LC analysis were of analytical grade or the highest available purity (Fisher Scientific, Illkirch, France). Deionized water was purified by a Milli-Q water-purification system (Millipore, Molsheim, France). All solutions prepared for HPLC were filtered through 0.45 µm nylon syringe membranes prior to use. SILM standards and methoxyflavone (internal standard) were purchased from Sigma (Saint Quentin Fallavier, France).

### 2.3. Liquid Chromatography Coupled with Mass Spectrometry (LC-MS) Analysis

All flavonolignans and taxifolin were quantified using a LC-MS analysis performed on a Water 2695 Alliance coupled with a single quadrupole mass spectrometer ZQ. LC-ESI-MS. Data acquisition and processing were performed with MassLynx 4.0 software (Waters-Micromass, Manchester, UK). The separation was performed as described in Drouet et al. (2018) [14] with slight modifications in the linear gradient: from a 10:90 (v/v) to 100:0 (v/v) mixture of A (methanol) and B (0.05% formic acid acidified water) respectively, at a flow rate of 1.00 mL/min.

### 2.4. Extraction

#### 2.4.1. Apparatus and General Procedure

1000 mg milled achene or whole fruit was extracted in 40 mL of aqEtOH solvent. The ultrasonic bath used was a USC1200TH (Prolabo, Fontenay-sous-Bois, France) with inner dimensions of 300 mm × 240mm × 200 mm and with a maximal heating power of 400 W (i.e., acoustic power of 1 W/cm^2^), equipped with a digital timer, a frequency and a temperature controller. The evaluation of extraction parameters was conducted using values ranging from 0 to 45 kHz for US frequency, 0 to 100% (v/v) for aqueous EtOH concentration, 15 to 60 min for extraction time, 30 °C to 70 °C for operating temperature, and 10:1 to 50:1 mL/g DW (DW: dried weight) for liquid to solid ratio. Prior to LC injection, the extract supernatant was filtered through 0.45 µm nylon syringe membranes. The optimized USAE method was compared with maceration in the same condition without application of US.

#### 2.4.2. Experimental Design

Following preliminary experiments, the full factorial design experiment and the resulting response surface plots were applied to identify the optimal extraction conditions for all flavonolignans using XLSTAT2019 software (Addinsoft, Paris, France). Variables were coded at three levels (−1, 0 and +1; Table 1). The three independent variables were EtOH concentration (X1 values were 50, 75 and 100%), US frequency (X2 values were 15, 30 and 45 kHz) and extraction time (X3 values were 20, 40 and 60 min) (Table 1). Here, twenty-seven batches were obtained by using the DOE (design of experiment) function of XLSTAT 2019 (Addinsoft, Paris, France), which take values of selective variables at different levels (Table 2). The experiments were carried out in triplicate. Equations of the models were calculated using the XLSTAT 2019 DOE analysis tool (Addinsoft, Paris, France). Surface plots showing the response as a function of the simultaneous variation of the independent variables were obtained with 3D option of XLSTAT 2019 (Addinsoft, Paris, France).

#### 2.4.3. Method Validation

The method precision, repeatability and stability were evaluated as described by Corbin et al. [15]. The precision, repeatability and stability were expressed in content (mg/g) and relative standard deviation (RSD, %).

### 2.5. Antioxidant Activity

#### 2.5.1. Cupric Ion Reducing Antioxidant Capacity (CUPRAC) Assay

Cupric ion reducing antioxidant capacity (CUPRAC) was used [32]. Briefly, 10 μL of an extract was mixed with 190 μL of the CUPRAC solution (composed of 10 mM Cu(II); 7.5 mM neocuproine and 1 M acetate buffer pH 7; ratio 1:1:1 (v/v/v)). Following incubation during 15 min at room temperature (25 ± 2 °C), the absorbance value at 450 nm of the reaction mixture was measured (BioTek ELX800; BioTek Instruments, Colmar, France).

#### 2.5.2. Inhibition of Advanced Glycation End Products (AGEs)

The inhibitory capacity of AGE formation was determined as described by Kaewseejan and Siriamornpun [33] using a 20 mg/mL BSA (Sigma Aldrich, Saint Quentin Fallavier, France) solution prepared in 0.1 M phosphate buffer (pH 7.4), a 0.5 M glucose (Sigma Aldrich, Saint Quentin Fallavier, France) solution prepared in phosphate buffer and a 0.1 M phosphate buffer at pH 7.4 containing 0.02% (w/v) sodium azide. Incubation was performed at 37 °C for five days in the dark. The amount of fluorescence resulting from the formation of AGEs was determined using 330 nm excitation wavelength and 410 nm emission wavelength conditions (VersaFluor fluorometer; Bio-Rad, Marnes-la-Coquette, France). The percentage of anti-AGEs formation was expressed as a % of inhibition relative to the control (addition of the same volume of extraction solvent).

### 2.6. Anti-Aging Activity

#### 2.6.1. Collagenase Assay

Collagenase from *Clostridium histolyticum* (Sigma Aldrich, Saint Quentin Fallavier, France) was used. The collagenase activity was determined using N-[3-(2-furyl) acryloyl]-Leu-Gly Pro-Ala (FALGPA; Sigma Aldrich, Saint Quentin Fallavier, France) as a substrate following the protocol of Wittenauer et al. [34]. Absorbance decrease was followed at 335 nm during 20 min thank to a microplate reader (BioTek ELX800; BioTek Instruments, Colmar, France). The collagenase activity in presence of each extraction conditions was determined in triplicated and the anti-collagenase activity was expressed, for each extract, as an inhibition percentage relative to corresponding control (adding same volume of extraction solvent).

#### 2.6.2. Elastase Assay

Elastase assay was performed by using porcine pancreatic elastase (Sigma Aldrich, Saint Quentin Fallavier, France). The elastase activity was determined using N-Succ-Ala-Ala-Ala-*p*-nitroanilide (AAAVPN; Sigma Aldrich, Saint Quentin Fallavier, France) as a substrate, as described by Wittenauer et al. [34]. The release of *p*-nitroaniline at 410 nm using a microplate reader (BioTek ELX800; BioTek Instruments). Triplicated measurements were performed, and the anti-elastase activity was expressed, for each extract, as an inhibition percentage relative to the corresponding control (adding same volume of extraction solvent).

### 2.7. Statistical Treatment of Data

The means and the standard deviations were used to present the data composed of three to five independent replicates. Student’s *t*-test was performed for comparative statistical analysis. Here, significant thresholds at *p* < 0.05, 0.01 and 0.001 were used for all statistical tests and represented by *, ** and ***, respectively. Model analysis (ANOVA) and 3D plots resulting from the combination of variables were conducted using XLSTAT 2019 and R (Addinsoft, Paris, France). The correlation values and corresponding *p*-values were obtained with Past 3.0 (Øyvind Hammer, Natural History Museum, University of Oslo, Oslo, Norway) by using the Pearson parametric correlation test.

## 3. Results

### 3.1. Preliminary Single Factor Experiments and Selection of Limiting Parameters

Several extraction parameters have been described to affect the extraction efficiency of polyphenols from various plant matrices [35]. Here, using a single-factor experiment approach, the influence of 5 independent parameters (aqEtOH concentration, extraction duration, US frequency, extraction temperature and liquid to solid ratio) on the SILM extraction yield from the mature fruit of *S. marianum* were evaluated. The objective of these preliminary experiments being to identify the limiting extraction parameters.

The choice of the solvent is an important parameter to fix during the development of an extraction method. Several organic solvents, such as methanol, ethanol (EtOH) or acetone, are commonly used for the extraction of plant polyphenols [36]. Here, considering our objective to propose these extracts for future cosmetic applications, and in consideration of the development of a green chemistry extraction method, EtOH was retained. Indeed, EtOH is a solvent that is less toxic to humans and more environmentally friendly when compared to other organic solvents (e.g., methanol) [35]. Moreover, its extraction capacity can be modulated by the addition of water, thus making it an ideal solvent for the extraction of a wide range of polyphenols with low to high polarity. Interestingly, these two universal solvents (i.e., EtOH and water) have commonly been used for various food and/or cosmetic applications [35,36].

**Figure 2:**
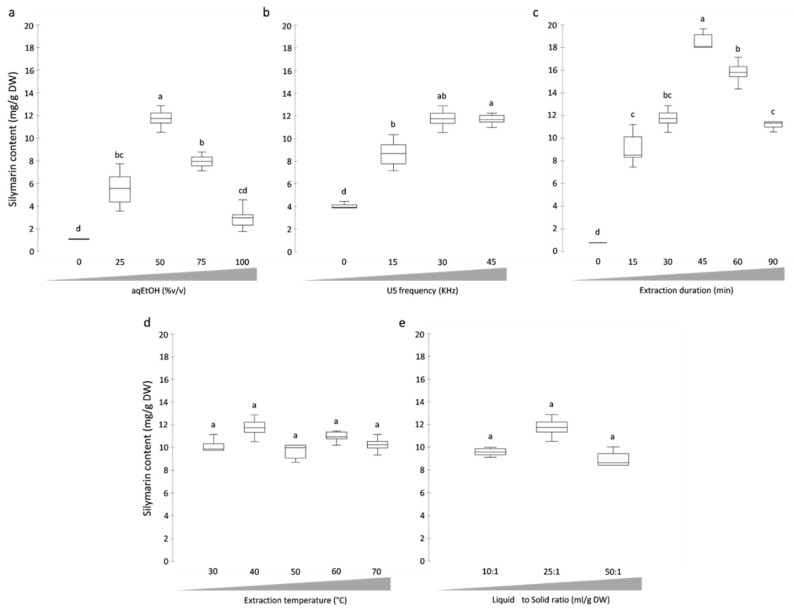
Silymarin (SILM) contents extracted from mature fruits of *S. marianum* as of function of (**a**) aqEtOH concentration (% (v/v)), (**b**) US frequency (kHz), (**c**) extraction duration (min), (**d**) extraction temperature (°C) and (**e**) liquid to solid ratio (mL/g DW). The complete description of each extraction conditions is presented in the text. Values are means ± SD of 6 independent replicates. Different letters (a–d) represent significant differences between the various extraction conditions (*p* < 0.05).

Here, the extraction capacity of 5 aqueous EtOH (aqEtOH) solutions at different concentrations (0%, 25%, 50%, 75%, and 100% (v/v) of EtOH in water) were assayed (Figure 2a). For these preliminary experiments evaluating the impact of aqEtOH concentrations, the other extraction parameters were arbitrary fixed to: 25:1 mL/g DW liquid to solid (L/S) ratio, 30 min for the extraction duration, 30 kHz for the US frequency and 45 °C for the extraction temperature. In our hands, aqEtOH concentration appeared to impact significantly the SILM extraction yield from milk thistle fruits. An optimal extraction yield was obtained for an aqEtOH concentration of 50% (v/v). Extreme values for aqEtOH concentrations (i.e., 0 and 100% (v/v)) resulted in 4- to 10-fold decreases in the SILM content, respectively, whereas aqEtOH concentrations of 25 and 75% (v/v) resulted in intermediate SILM contents.

The US frequency is also known to potentially impact the extraction efficiency. This parameter acts through the modulation of the cavitation effect and the diffusion coefficient of the targeted compounds into the extraction solvent. This could result in a greater solubilization of the target compound in the considered extraction solvent, and to a higher extraction efficiency [36]. Increasing the US frequency could result in a lower extraction duration, and therefore a lower energy consumption [37]. However, application of high US frequencies could alter or destroy the native structure, thus reducing both the extraction yield and the biological activity of the targeted compound(s) [38]. Consequently, US frequency has to be considered carefully during the development of an UAE method. In our hands, the impact of 4 different US frequencies (0, 15, 30 and 45 kHz) on the SILM extraction yield was evaluated (Figure 2b). For this purpose, the other extraction parameters were arbitrary fixed to: 50% (v/v) aqEtOH concentration, 25:1 mL/g DW L/S ratio, 30 min for the extraction duration and 45 °C extraction temperature. We noted a significant impact of US frequency, with the highest extraction yield obtained using 30 kHz US frequency. The absence (0 kHz) or lower application (15 kHz) of US frequency resulted in a lower extraction efficiency, whereas the highest applied US frequency (45 kHz) led to a decrease in the SILM extraction yield, possibly because of the reported destructive effect high US frequencies [38].

To reduce energy consumption in a context of a green chemistry approach, optimizing extraction duration is essential [37]. Increasing extraction duration will not necessarily result in a gain in terms of extraction yield, since a prolonged US exposure can lead to the deterioration of the compounds [38]. Here, we considered 6 extraction durations (0, 15, 30, 45, 60 and 90 min) with the other parameters arbitrarily fixed to: 50% (v/v) aqEtOH concentration, 25:1 mL/g DW L/S ratio, 30 kHz US frequency duration and 45 °C extraction temperature. A gradual increase in SILM extraction yield from milk thistle fruit as a function of the extraction duration was first observed. The maximum extraction efficiency was reached after 45 min, followed by a significant decrease with 60- and 90-min extraction time (Figure 2c). This observation is in agreement with other studies reporting on the degradation of antioxidant phenolic compounds with prolonged US treatments [35,37,38].

Different extraction temperatures (30, 40, 50, 60 and 70 °C) were next evaluated (Figure 2d). The other parameters were fixed to: aqEtOH concentration 50% (v/v), L/S ratio 25:1 mL/g DW, extraction time 45 min and US frequency 30 kHz. Using these conditions, the extraction temperature was not identified as a limiting parameter. According to the hot spot theory, the cavitation bubbles are considered as a microreactor generating a local environment in the surrounding liquid after their collapse with high temperature (ca. 4500 °K) and pressure (ca. 1000 atm) [36]. This theory could explain the low impact of few dozen temperature degrees on the SILM extraction yield. This parameter was not considered as a limiting parameter, and was not further optimized. An extraction temperature of 45 °C was used hereafter.

Finally, 3 liquid to solid (L/S) ratios (10:1, 25:1 and 50:1, in mL of aqEtOH (50% (v/v)) per gram of DW material) were evaluated (using fixed aqEtOH concentration of 50% (v/v), extraction duration of 30 min, US frequency of 30 kHz and extraction temperature of 45 °C) (Figure 2e). Only slight and non-significant differences in SILM extraction yields were observed. This parameter was not considered as s limiting parameter, and was not further optimized. Slightly better results were obtained with a L/S ratio of 25:1, and therefore this was used hereafter.

### 3.2. Development of a Multifactorial Approach

From the preliminary experiments, the significant impacts of aqEtOH concentration, extraction duration and US frequency were evidenced (Figure 2). These parameters were therefore selected for further optimization. To take into account the possible interactive influence of these parameters, experimental factorial design (design of experiment, DOE) coupled with statistical analysis was employed. This strategy is known to be more effective, precise and rapid for integrating a large number of extraction conditions and for evidencing possible interactions between independent variables as compared with single factor approaches [39]. Taking into account the preliminary experiments, the 3 influencing variables were: aqEtOH concentration (variable X_1_, ranging from 25 to 75% (v/v)), US frequency (variable X_2_, ranging from 15 to 45 kHz) and extraction duration (variable X_3_, ranging from 20 to 60 min). Their coded levels and experimental values are presented in Table 1. According to the results obtained during preliminary experiments for L/S ratio and extraction temperature, these parameters were fixed to 25:1 mL/g DW and 45 °C, respectively.

**Figure 3:**
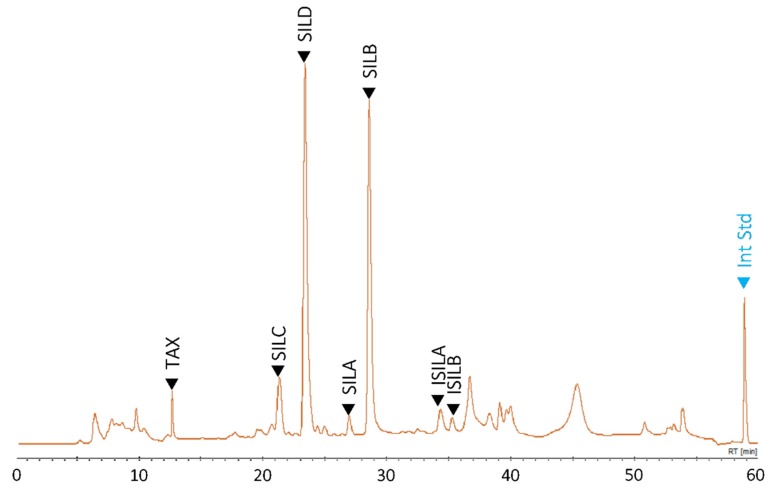
Representative HPLC chromatogram of an extract prepared by UAE of a mature fruits (achenes) of a milk thistle (*S. marianum*) commercial cultivar. The main compounds considered in this study are the flavonoid taxifolin (TAX) and the flavonolignans: silychristin (SILC), silydianin (SILD), silybin A (SILA), silybin B (SILB), isosilybin A (ISILA) and isosilybin B (ISILB). Int Std: internal standard (6-methoxyflavone).

Full factorial design was used to optimize this extraction process considering its high reproducibility as a consequence of the real measurement of a large number of experimental conditions compared to other DOE approaches [40]. The 27 different bath conditions (run ID) were determined and randomized (run order) in silico. The corresponding independent process variables of each batch condition are presented in Table 2. Each batch condition was assayed in independent triplicates. The separation was based on the method described by Drouet et al. [14], here further optimized (see Materials and Methods, Section 2.3), allowing a high resolutive separation of the different peaks as shown in Figure 3. The extraction yield results for SILM and each individual constituent of this mixture are presented in Table 2 and Table S1, respectively.

The SILM content extracted from mature fruit of *S. marianum* ranged from 1.80 (run ID#13) to 17.98 (run ID#26) mg/g DW (Table 2).

The individual composition of the SILM of each extract was also determined (Table S1), with results given from the most to the less abundant component:

- SILB was detected under each extraction condition and its contents ranged from 1.29 (run ID#1) to 7.52 (run ID#26) mg/g DW;
- the detected SILD contents ranged from 0.40 (run ID# 13) to 4.21 (run ID#20) mg/g DW, whereas SILD was not detected for one extraction condition (run ID#16). This run ID#16 presented low aqEtOH concentration (25% (v/v)) and high US frequency (45 kHz);
- the detected ISILA contents ranged from 0.45 (run ID#3) to 2.49 (run ID#26) mg/g DW, whereas ISILA was not detected for 9 extraction conditions (run ID#1, #4, #7, #10, #13, #16, #19, #22 and #25). All these run IDs presented the lowest aqEtOH concentration (i.e., 25% (v/v)) as X_1_ condition (i.e., X_1_ = −1, Table 1);
- the detected SILC contents ranged from 0.01 (run ID#19) to 1.52 (run ID#26) mg/g DW, whereas SILC was not detected for 4 extraction conditions (run ID#7, #16, #22 and #25). Runs #7, #16 and #25 presented low aqEtOH concentration (25% (v/v), X_1_ = −1, Table 1) and high US frequency of 45 kHz (X_2_ = +1, Table 1). Run ID#22 presented the same low aqEtOH concentration and intermediate US frequency of 30 kHz (X_2_ = 0, Table 1) but during a prolonged period of time (X_3_ = +1 (i.e., 60 min), Table 1);
- the detected SILA contents ranged from 0.01 (run ID#13) to 1.09 (run ID#26) mg/g DW, whereas SILA was not detected for 3 extraction conditions (run ID#4, #16 and #24). These run IDs presented the same low aqEtOH concentration (i.e., 25% (v/v), X_1_ = −1, Table 1), whereas run ID#16 and #24 presented high US frequency (X_2_ = +1, Table 1) and prolonged extraction duration of 40 and 60 min, respectively (X_3_ = 0 or +1, respectively, Table 1);
- TAX was detected under each extraction conditions and its content ranged from 0.16 (run ID#3) to 0.68 (run ID#20);
- and finally, the detected ISILB contents ranged from 0.03 (run ID#9) to 0.55 (run ID#26) mg/g DW, whereas ISILB was not detected for 3 extraction conditions (run ID#1–4, #6, #7, #10, #12–16, #19, #22, #24, #25 and #27). Here, we therefore observed that the use of aqEtOH concentration of 25% (v/v) failed to extract ISILB. These results may also be related to the low accumulation of ISILB in the mature fruit of the considered milk thistle cultivar.

To sum up these results, the hypothesis of low extraction yields of SILM and its constituents as a consequence of their lower solubility in extraction solvent with high polarity (i.e., 25% (v/v) aqEtOH concentration, X_1_ = −1, Table 1) and/or of drastic/destructive extraction conditions (i.e., high and/or prolonged US treatment) can be made.

A model of the SILM extraction yield as a function of the 3 different variables was obtained by multiple regression analysis (Table 3). Using the conditions described in Table 1 and Table 2, the SILM extraction yield (Y_SILM_) as a function of the 3 different variables (X_1_: aqEtOH concentration, X_2_: US frequency and X_3_: extraction duration) in the form of a polynomial equation was: Y_SILM_ = 13.52 + 2.29X_1_ + 0.78X_2_ + 1.96X_3_ − 7.45X_1_^2^ − 0.86X_2_^2^ − 0.25X_3_^2^ + 0.32X_1_X_2_ + 0.55X_1_X_3_ − 0.11X_2_X_3_ (Table 3).

The statistical analysis (Table 3) evidenced the significant impact on the SILM extraction efficiency from mature fruit of *S. marianum* of the linear coefficients X_1_ (aqEtOH concentration) and X_2_ (extraction time) and the quadratic coefficients X_1_^2^. On the contrary, the other linear X_3_ (US frequency), quadratic X_2_^2^ and X_3_^2^ as well as interaction coefficients were not significant (*p* > 0.05). Therefore, aqEtOH concentration (X_1_), as well as extraction duration (X_3_), appeared to be the most influential parameters for this extraction process over US frequency (X_2_) for SILM extraction. The same trend was observed for the individual constituents of the SILM, with the exception of ISILB for which the quadratic coefficients X_1_^2^ was the sole significant coefficient (Table S2).

In addition to all these significant coefficients, SILB extraction was also significantly impacted by the US frequency (linear coefficients X_2_). SILB was therefore the only compound for which extraction was significantly influenced by this US frequency variable. Nevertheless, we have to keep in mind, here, that during the DOE under all the extraction conditions, US were applied at 3 different frequencies that appeared to be in the best range in preliminary experiments. From these preliminary experiments, it clearly appeared that the absence of US treatment drastically reduced extraction efficiency. Therefore, here we can conclude that US frequency X_3_ variable did not significantly influenced the SILM extraction yield in the selected range of values for this variable, whereas the absence of US had clearly resulted in a less efficient extraction process during the preliminary experiments.

Results of the analysis of variance (ANOVA) and the fit for the models obtained for SILM and its constituents are listed in Table 4 and Table S3, respectively. The high F-value (14.73) and the low *p*-value (*p* < 0.0001) indicated that the model was highly significant and could predict the SILM content as a function of the variable values with a great precision (Table 4). The same trend was recorded for each individual constituent of the SILM (Table S3), with a lower but still significant precision for ISILB. This was also confirmed by the low and non-significant lack of fit values. The model precision in the prediction of the experimental values is evidenced by the predicted vs. experimental plot presented in Figure S1. A determination coefficient *R^2^* of 0.891 (with adjusted value of 0.833) for SILM extraction model, and ranging from 0.810 for TAX to 0.946 both for SILC and SILD extraction models were obtained. ISILB extraction models presented the lowest *R^2^* value of 0.589 (Table 4 and Table S3). The coefficient value (CV) indicated the adequacy between the model and experimental values.

To understand the complexity of the models, 3D plots were drawn for SILM (Figure 4) and each individual constituent (Figures S2–S8).

**Figure 4:**
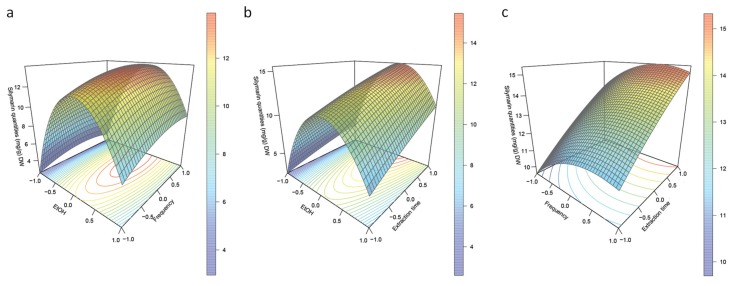
3D plots from the model predicted SILM extracted quantities from mature fruits of *S. marianum* as a function of (**a**) aqEtOH concentration and US frequency, (**b**) aqEtOH concentration and extraction duration, and (**c**) US frequency and extraction duration.

The linear coefficients of the second-order polynomial equation for X_1_ aqEtOH concentration, X_2_ US frequency and X_3_ extraction duration, as well as the interaction coefficients X_1_X_2_ (aqEtOH concentration x US frequency) and X_1_X_3_ (aqEtOH concentration x extraction duration), were all positives, indicating that the increase of these parameters results in a favourable action on the SILM extraction yield. However, their low values, in association with the negative values recorded for their quadratic coefficients (X_1_^2^, X_2_^2^ and X_3_^2^, respectively), as well as for the interaction coefficient between aqEtOH concentration and US frequency (X_2_X_3_), indicate that the extraction of SILM reaches a maximum value before decreasing for high values of these parameters. We can observe these tendencies on the 3D plots with first a positive action on SILM extraction yield with increased aqEtOH concentrations combined with higher US frequency and/or prolonged extraction duration (Figure 4a,b). However, the highest aqEtOH concentration, on the one hand, as well as prolonged extraction duration at high US frequency, on the other hand, resulted in a marked decline of SILM extraction yields (Figure 4). Ethanol/water mixtures represent commonly used eco-friendly solvents able to extract a wide range of polyphenols; however, optimal aqEtOH concentration is highly dependent of the nature of the considered polyphenol [36]. Applying high US frequency during a prolonged period of time is known to be potentially destructive and to induce polyphenols oxidation, especially when water is used as solvent [27,36,38,41]. This could lead to a significant decrease in extraction yield, but also to the loss of the biological activities of the extract [27,36,38,41].

Here, according to the adjusted second order polynomial equation, optimal conditions were: 54.5% (v/v) aqEtOH as solvent, 36.6 kHz for the US frequency and 60 min as extraction time (with a fixed extraction temperature of 45 °C and liquid to solid ratio of 25:1 mL/g DW). Adjusted to the material, an US frequency of 30 kHz was used. Under these optimized conditions, SYLM extraction yield from the mature fruit of AJQ *S. marianum* cultivar reached 20.28 ± 0.41 mg/g DW.

### 3.3. Validation of the Extraction Method

As shown in Figure 3, the identification and quantification of the SILM different constituents were using the validated separation method described by Drouet et al. [14] by comparison with an authentic commercial standards, and further confirmed by LC-MS. Here, the separation resolution was further ameliorated following slight modification of the mobile phase (see Materials and Methods, Section 2.4), thus allowing a precise quantification of each compound. Coupled with the present optimized extraction method, in order to certify accuracy and precision, the method was then validated according to the recommendations of the association of analytical communities (AOAC) (http://www.aoac.org). The parameter values of this validation procedure are satisfactory in terms of precision, repeatability and stability according to AOAC standards and are presented in Table 5.

### 3.4. Evaluation of the Biological Activities of the Extracts Relevant to Cosmetics

To evaluate the influence of the extraction process, to ensure that the biological activity is retained during this process, and to correlate the biological activity with the phytochemical profiles of the extracts, we next determined the antioxidant and anti-aging potential relevant to cosmetics of each of the 27 run IDs. Indeed, SILM and its flavonolignans have received a recent interest for their potent antioxidant and anti-aging activities relevant to cosmetic [14,15,16,17,18,19].

**Figure 5:**
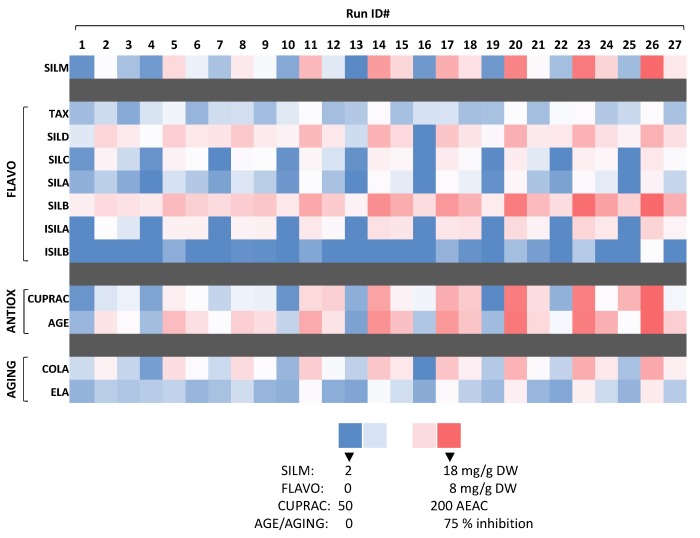
Heatmap showing the phytochemical composition and biological activities relevant to cosmetic of the 27 extracts from *S. marianum* mature fruit following UAE. Two antioxidant assays were conducted: CUPRAC (expressed as ascorbic acid equivalent antioxidant capacity (AEAC, in µM AEAC)) and the inhibition of advanced glycation end product (AGE) formation (expressed in % of inhibition relative to a control obtained by measuring the activity of the corresponding extraction solvent). Two anti-aging assays were conducted by determining the inhibition activity of each extracts toward collagenase (COL) and elastase (ELA) enzymes (expressed in % of inhibition relative to a control obtained by measuring the activity of the corresponding extraction solvent).

CUPRAC assay has been reported to effectively evidence the antioxidant activity of milk thistle extracts [20]. Here, the antioxidant activity evaluated by the CUPRAC assay ranged from 51.33 (run ID#10—SILM content of 3.21 mg/g DW) to 183.80 (run ID#26—SILM content of 17.98 mg/g DW) µM AEAC (Figure 5, Table S4). Oxidative stress has been associated with aging and could lead to the formation of advanced glycation end products (AGEs) [42]. Here, the strong inhibition of AGEs formation confirmed the antioxidant capacity of these extract evidenced by the CUPRAC assay. The inhibition of AGEs formation ranged from 6.64 (run ID#13—SILM content of 1.80 mg/g DW) to 74.31 (run ID#26—SILM content of 17.98 mg/g DW) % of inhibition (Figure 5, Table S4). A strong significant correlation was observed between SILM content and CUPRAC antioxidant activity (PCC = 0.862) as well as between SILM content and AGEs inhibitory action (PCC = 0.997) (Table 6).

All the SILM constituents were also correlated with these antioxidant activities (Table 6). The SILM content and composition of wild ecotypes of *S. marianum* from Pakistan have been correlated with their antioxidant activity measured by CUPRAC assay [14]. Natural antioxidants have attracted growing interest over the last decade because of their possible use as alternative to the potentially harmful, synthetic antioxidant such as butylated hydroxyanisole (BHA) or butylated hydroxytoluene (BHT) in different formulations [43,44,45]. Recently, a SILB palmitate derivative has been synthesized and displayed a pronounced anti-lipoperoxidant activity, inhibiting the formation of conjugated diene production in two different lipophilic media (bulk oil and o/w emulsion) subjected to accelerated storage test [45]. Here, this antioxidant action in vitro is further confirmed by the CUPRAC assay correlated with the SILM and SILB contents. In addition, oxidative stress has been associated with aging and age-related diseases [46], in particular leading to the formation of AGE [47]. The ability of natural compounds to inhibit their formation have therefore attracted increasing attention in cosmetics. The high inhibition of AGE formation also correlated with the SILM content, in particular with SILA and SILB which is of special interest for future applications.

The next step was to evaluate the inhibitory activity of the extracts toward collagenase and elastase. Indeed, the potent inhibitory action of SILM and its flavonolignans toward these enzymes has been recently evidenced [15]. A strong inhibitory effect was observed for collagenase, whereas it was less marked for elastase (Figure 5, Table S4). Collagenase inhibition ranged from 4.21 (run ID#16—SILM content of 2.49 mg/g DW) to 49.13 (run ID#26—SILM content of 17.98 mg/g DW) % of inhibition, while, elastase inhibition ranged from 6.84 (run ID#13—SILM content of 1.80 mg/g DW) to 22.93 (run ID#26—SILM content of 17.98 mg/g DW) % of inhibition. A strong and significant correlation linking these enzymatic inhibitory actions with SILM content was measured (Table 6). Elastase and collagenase enzymes act on the remodelling and/or degradation of the extracellular matrix components in the dermis, thus potentially leading to skin alterations such as skin tonus decrease, formation of deep wrinkles and resilience losses [48,49,50]. Our results confirmed the potential of SILM and its constituent as inhibitor of collagenase, and to a less extend of elastase. Future works focusing on the inhibition mechanism rationalization of each flavonolignans would be of particular interest for future applications.

### 3.5. Comparison with Conventional Maceration Protocol

To evaluate its efficiency, the present optimized green US extraction procedure was compared with a conventional heat reflux extraction method. For this purpose, we used the same aqEtOH concentration of 54.5% (v/v), extraction duration of 60 min, temperature of 45 °C and L/S ration of 25:1. The sole difference between the two extraction methods was the US frequency: with application of US frequency at 30 kHz for the present optimized UAE extraction procedure, while no US was applied for the conventional maceration protocol operating in a classical water bath. The results of these extractions are presented in Table 7.

The results of these two different extraction processes demonstrated that application of US frequency at 30 kHz using our UAE protocol resulted in a significant gain (ca. 6 fold) in SILM content as compared to maceration. The gains in the extraction yields of the SILM constituents ranged from 2.56 for SILC to 40.37 for ISILB (Table 7). Please note that higher extraction yields were obtained when increasing the extraction duration of maceration, but still without reaching values observed with the UAE (data not shown). This protocol is therefore of special interest, in the context of green chemistry, in terms of reducing energy consumption by using this innovative technology, but also for industrial processes. It allows high extraction yields of milk thistle flavonolignans with reduced extraction costs (reduction in terms of treatment duration and solvent consumption). This efficiency of UAE could be a consequence of the hot spot hypothesis: cavitation bubbles acting as a microreactor generating a high temperature and pressure local environment in the surrounding liquid after their collapse resulting in a more efficient rupture of the plant tissue, and therefore a more efficient release and solubilization of the phytochemicals [36].

### 3.6. Comparison of the SILM Variations in Established Cultivars vs Wild Ecotypes

Taking advantage of this optimized and validated UAE protocol, we applied it to compare the content and composition of SILM of 4 established cultivars. The results of these extractions are presented in Table 8.

AJN cultivar is the richest in SILM contents, and accumulated the highest contents in SILC, SILD and SILA, whereas AJQ was the richest in SILB, ISILA and ISILB. The highest accumulation in TAX was measured in 11E. Here, we observed quite restricted variation ranges compared to our previous study with wild ecotypes from Pakistan [14]. It is accepted that the SILM content and composition could vary according to both genetic background and culture conditions [14,51,52,53]. Strong variations in SILM content and composition was reported for wild ecotypes from Egypt [54], Iran [52], and Greece [55], as well as from Poland, Hungary, Bulgaria, and Germany [51]. Culture conditions of the commercial crop are probably more homogenous than natural conditions which could partly explain this led wide range of contents. Here the observed stability in the SILM contents and composition is an important feature for these established cultivars cultivated for commercial purposes. Here, the same culture conditions were used for the 4 analysed cultivars. However, the information on the wide range of variations observed in wild ecotypes could be relevant for the generation of new cultivars in future breeding strategies for more specific applications.

## 4. Conclusions

*Silybum marianum* (L.) Gaertn., the so-called milk thistle, constitutes a unique source of silymarin (SILM), and thus is an attractive starting material for their extraction. SILM is a mixture of flavonolignans accumulated in the mature fruits of *S. marianum*. These compounds have attracted a recent interest for cosmetic applications, and therefore deserve the development of optimized and validated green extraction process. Here, we developed an ultrasound-assisted extraction (UAE) of SILM from mature fruits of *S. marianum* using a design of experiment strategy. The optimal conditions for UAE were: aqEtOH 54.5% (v/v) as solvent, US frequency 36.6 kHz and extraction time 60 min, with temperature fixed at 45 °C and liquid to solid ratio of 25:1 mL/g DW. Following its optimization, this extraction method was validated according to international standards of the association of analytical communities (AOAC) to ensure its precision and accuracy in the quantitation of the individual SILM constituents. The efficiency of UAE allowed substantial gains in terms of SILM extraction yield compared to conventional extraction by maceration. The UAE allows an efficient extraction in a reduced extraction time. Thus, the present method is of particular interest in the context of green chemistry in terms of reducing energy consumption and with the use of a green solvent. High antioxidant capacity of the extracts was evidenced by the in vitro assays CUPRAC and inhibition of advanced glycation end products (AGEs). The skin anti-aging action was also confirmed by the strong in vitro cell-free inhibition capacity of the obtained extract against collagenase and elastase enzymes.

To resume, the procedure presented here allows a green efficient extraction of SILM flavonolignans from the fruits of *S. marianum* with potent antioxidant and anti-aging activities. Altogether, these results prove that the US extraction method presented here resulted in high extraction capacity of SILM and its constituents, but also that the native biologically active forms of these compounds is retained during extraction. We anticipate and suggest that further analysis of the cytotoxicity of the extract should be perform, in order to allow this fast, easy efficient and reproducible extraction method of these compounds to be employed in the real practical cosmetic product development.

## Figures and Tables

**Table 1:** Identity, code unit, coded level and experimental values of the 3 independent variables.

Independent Variable	Code Unit	Coded Variable Levels		
		−1	0	+1
aqEtOH concentration (% v/v) ^1^	X_1_	25	50	75
US frequency (kHz)	X_2_	15	30	45
Extraction duration (min)	X_3_	20	40	60

^1^ % of aqEtOH concentration in mixture with HPLC grade ultrapure water.

**Table 2:** Results of full factorial design experiments for the extraction of silymarin (SILM) from mature fruits of *S. marianum*.

Run ID	Run Order	X_1_	X_2_	X_3_	SILM (mg/g DW)
Run ID#1	17	−1	−1	−1	2.24 ± 1.80
Run ID#2	24	0	−1	−1	6.99 ± 4.11
Run ID#3	26	+1	−1	−1	4.33 ± 3.09
Run ID#4	21	−1	0	−1	2.55 ± 4.61
Run ID#5	22	0	0	−1	9.66 ± 1.44
Run ID#6	6	+1	0	−1	6.51 ± 3.06
Run ID#7	10	−1	+1	−1	4.37 ± 1.72
Run ID#8	27	0	+1	−1	8.45 ± 4.48
Run ID#9	7	+1	+1	−1	6.88 ± 4.82
Run ID#10	18	−1	−1	0	3.21 ± 0.34
Run ID#11	12	0	−1	0	12.12 ± 1.57
Run ID#12	8	+1	−1	0	6.15 ± 1.08
Run ID#13	25	−1	0	0	1.80 ± 1.50
Run ID#14	1	0	0	0	14.25 ± 0.51
Run ID#15	16	+1	0	0	9.79 ± 2.47
Run ID#16	23	−1	+1	0	2.49 ± 1.59
Run ID#17	11	0	+1	0	12.88 ± 3.35
Run ID#18	14	+1	+1	0	9.13 ± 1.80
Run ID#19	15	−1	−1	+1	2.49 ± 0.33
Run ID#20	3	0	−1	+1	16.00 ± 0.35
Run ID#21	13	+1	−1	+1	7.27 ± 0.36
Run ID#22	9	−1	0	+1	4.20 ± 0.30
Run ID#23	5	0	0	+1	16.71 ± 0.19
Run ID#24	19	+1	0	+1	10.05 ± 0.48
Run ID#25	4	−1	+1	+1	4.04 ± 0.30
Run ID#26 *	20	0	+1	+1	17.98 ± 0.66
Run ID#27	2	+1	+1	+1	8.54 ± 0.94

Values are the mean ± RSD of 3 independent replicates except for *, which correspond to the highest SILM content here determined by 6 independent experiments to confirm this value.

**Table 3:** Values, standard deviations and statistical analysis of the regression coefficients for the SILM extraction yield (Y_SILM_) from mature fruits of *S. marianum* as a function of the 3 different variables (X_1_: aqEtOH concentration, X_2_: US frequency and X_3_: extraction duration).

Source	Value	SD	*t*	*p* > |*t*|
Constant	13.52	1.0	13.75	<0.0001 ***
X_1_	2.29	0.5	5.04	0.0001 ***
X_2_	0.78	0.5	1.70	0.11 ^ns^
X_3_	1.96	0.5	4.31	0.0004 ***
X_1_^2^	−7.45	0.8	−9.44	<0.0001 ***
X_2_^2^	−0.86	0.8	−1.09	0.29 ^ns^
X_3_^2^	−0.25	0.8	−0.31	0.76 ^ns^
X_1_X_2_	0.32	0.6	0.58	0.57 ^ns^
X_1_X_3_	0.55	0.6	0.98	0.34 ^ns^
X_2_X_3_	−0.11	0.6	−0.20	0.84 ^ns^

SD standard error; *** significant *p* < 0.001; ** significant *p* < 0.01; * significant *p* < 0.05; ns not significant.

**Table 4:** ANOVA of the SILM extraction model.

Source	Sum of Square	df	Mean of Square	*F*-Value	*p*-Value
Model	517.0	9	57.4	15.4	<0.0001
Lack of fit	63.4	17	3.7	0.060	ns
Residual	58.5	17	3.4	-	-
Pure Error	4.9	0	-	-	-
Cor. Error	580.4	26	-	-	-
*R^2^*	0.891				
adj *R^2^*	0.833				
CV %	0.238				

df: degree of freedom; Cor. Error: corrected error; *R^2^*: determination coefficient; *R^2^* adj: adjusted *R^2^*; CV variation coefficient value; ns: non-significant.

**Table 5:** Precision, repeatability and stability validation parameters of the method.

Compound	Precision (*n = 5*)		Repeatability (*n = 5*)		Stability (*n = 5*)	
	*Content mg/g DW*	*RSD (%)*	*Content mg/g DW*	*RSD (%)*	*Content mg/g DW*	*RSD (%)*
SILM	20.28 ± 2.02	2.02	19.12 ± 0.88	4.62	20.16 ± 0.43	2.16
SILC	1.93 ± 0.06	3.00	1.71 ± 0.10	5.72	2.02 ± 0.07	3.40
SILD	2.40 ± 0.13	5.57	2.63 ± 0.08	3.20	2.56 ± 0.10	3.94
SILA	1.06 ± 0.03	3.11	1.02 ± 0.02	2.47	1.17 ± 0.04	3.68
SILB	8.43 ± 0.13	1.54	7.96 ± 0.28	3.58	8.17 ± 0.33	4.02
ISILA	4.17 ± 0.14	3.24	3.89 ± 0.36	9.25	4.14 ± 0.11	2.64
ISILB	2.29 ± 0.13	5.48	1.91 ± 0.15	7.83	2.10 ± 0.07	3.26

RSD: relative standard deviation (expressed in %).

**Table 6:** Pearson coefficient correlation (PCC) linking SILM and its constituents to the in vitro antioxidant and in vitro cell-free anti-aging activities.

Assay	SILM	TAX	SILC	SILD	SILA	SILB	ISILA	ISILB
CUPRAC	0.862 ***	0.500 *	0.768 *	0.776 *	0.806 *	0.887 *	0.827 **	0.697 *
AGE	0.997 ***	0.604 *	0.930 **	0.942 **	0.976 *	0.979 ***	0.960 **	0.766 *
COLA	0.976 **	0.659 **	0.957 **	0.927 **	0.968 **	0.928 ***	0.908 **	0.801 *
ELA	0.922 **	0.702 **	0.893 **	0.843 *	0.910 **	0.894 *	0.830 *	0.843 *

*** significant *p* < 0.001; ** significant *p* < 0.01; * significant *p* < 0.05.

**Table 7:** Comparison between conventional the present optimized ultrasound-assisted extraction (UAE) vs conventional heat reflux method (MAC).

Compound	UAE	Maceration (MAC)	Ratio UAE/MAC
SILM	20.28 ± 0.41	3.40 ± 0.14	5.96 ***
SILC	2.40 ± 0.13	0.94 ± 0.04	2.56 ***
SILD	1.93 ± 0.06	0.68 ± 0.04	2.84 ***
SILA	1.06 ± 0.03	0.11 ± 0.02	9.32 ***
SILB	8.43 ± 0.13	1.31 ± 0.04	6.41 ***
ISILA	4.17 ± 0.13	0.30 ± 0.01	13.84 ***
ISILB	2.29 ± 0.12	0.06 ± 0.01	40.37 ***

Values are the mean ± RSD of three independent replicates expressed in mg/g DW. *** indicate significant differences (*p* < 0.001) between conditions.

**Table 8:** Contents of SILM constituents of 4 French milk thistle cultivars.

Cultivars	SILM	TAX	SILC	SILD	SILA	SILB	ISILA	ISILB
APM	35.40 ± 1.31	1.27 ± 0.18	1.79 ± 0.32	9.67 ± 1.45	6.10 ± 0.40	5.30 ± 1.37	1.40 ± 0.10	0.67 ± 0.25
AJN	**43.61 ± 1.61**	1.58 ± 0.13	**3.19 ± 0.64**	**11.77 ± 1.77**	**7.39 ± 1.10**	6.46 ± 1.67	1.23 ± 0.27	0.63 ± 0.10
AJQ	20.28 ± 0.41	0.82 ± 0.05	2.40 ± 0.13	1.93 ± 0.06	1.06 ± 0.03	**8.43 ± 0.13**	**4.17 ± 0.13**	**2.29 ± 0.12**
11E	43.25 ± 1.60	**1.62 ± 0.11**	2.14 ± 0.10	11.35 ± 1.71	6.78 ± 1.56	7.43 ± 1.92	1.65 ± 0.30	0.60 ± 0.16

Values are the mean ± RSD of three independent replicates expressed in mg/g DW. Maximum contents are in bold.

## Supplementary Materials

The following are available online at https://www.mdpi.com/2076-3921/8/8/304/s1, **Figure S1** Biplot representation of the linear relation between predicted *vs* measured SILM contents in the 27 sample extracts. Light blue contours represented *p* = 0.05. **Figure S2** 3D plots from the model predicted TAX extracted quantities from mature fruits of *S. marianum* as a function of (a) ethanol concentration and ultrasound frequency, (b) ethanol concentration and extraction duration, and (c) ultrasound frequency and extraction duration. **Figure S3** 3D plots from the model predicted SILC extracted quantities from mature fruits of *S. marianum* as a function of (a) ethanol concentration and ultrasound frequency, (b) ethanol concentration and extraction duration, and (c) ultrasound frequency and extraction duration. **Figure S4** 3D plots from the model predicted SILD extracted quantities from mature fruits of *S. marianum* as a function of (a) ethanol concentration and ultrasound frequency, (b) ethanol concentration and extraction duration, and (c) ultrasound frequency and extraction duration. **Figure S5** 3D plots from the model predicted SILA extracted quantities from mature fruits of *S. marianum* as a function of (a) ethanol concentration and ultrasound frequency, (b) ethanol concentration and extraction duration, and (c) ultrasound frequency and extraction duration. **Figure S6** 3D plots from the model predicted SILB extracted quantities from mature fruits of *S. marianum* as a function of (a) ethanol concentration and ultrasound frequency, (b) ethanol concentration and extraction duration, and (c) ultrasound frequency and extraction duration. **Figure S7** 3D plots from the model predicted ISILA extracted quantities from mature fruits of *S. marianum* as a function of (a) ethanol concentration and ultrasound frequency, (b) ethanol concentration and extraction duration, and (c) ultrasound frequency and extraction duration. **Figure S8** 3D plots from the model predicted ISILB extracted quantities from mature fruits of *S. marianum* as a function of (a) ethanol concentration and ultrasound frequency, (b) ethanol concentration and extraction duration, and (c) ultrasound frequency and extraction duration. **Table S1** Results of full factorial design experiments for the extraction of TAX, SILC, SILD, SILA, SILB, ISILA and ISILB from mature fruits of *S. marianum*. **Table S2** Values, standard deviations and statistical analysis of the regression coefficients for the TAX, SILC, SILD, SILA, SILB, ISILA and ISILB extraction yield from mature fruits of *S. marianum* as a function of the 3 different variables (X1: ethanol concentration, X2: ultrasound frequency and X3: extraction duration). **Table S3** ANOVA results of the TAX, SILC, SILD, SILA, SILB, ISILA and ISILB extraction models. **Table S4** Individual antioxidant and anti-aging activities of the 27 US extract samples.
