# Supplementary material for: A Green Ultrasound-Assisted Extraction Optimization of the Natural Antioxidant and Anti-Aging Flavonolignans from Milk Thistle Silybum marianum (L.) Gaertn. Fruits for Cosmetic Applications
Source: Antioxidants (Basel). 2019 Aug 14;8(8):304. doi: 10.3390/antiox8080304 (PMC6721202; doi:10.3390/antiox8080304)
Supplement: Supplementary file 1 [file antioxidants-08-00304-s001.pdf]

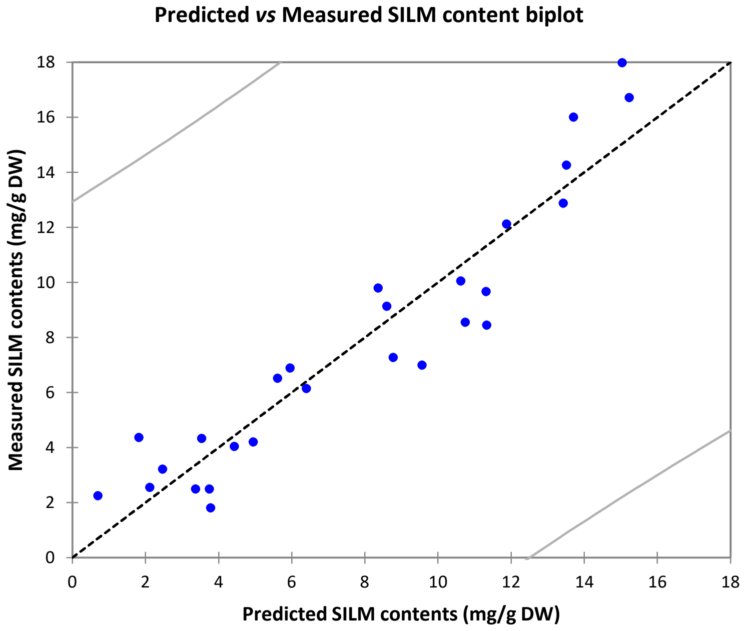

Figure S1 Biplot representation of the linear relation between predicted *vs* measured SILM contents in the 27 sample extracts. Light blue contours represented $p = 0.05$.

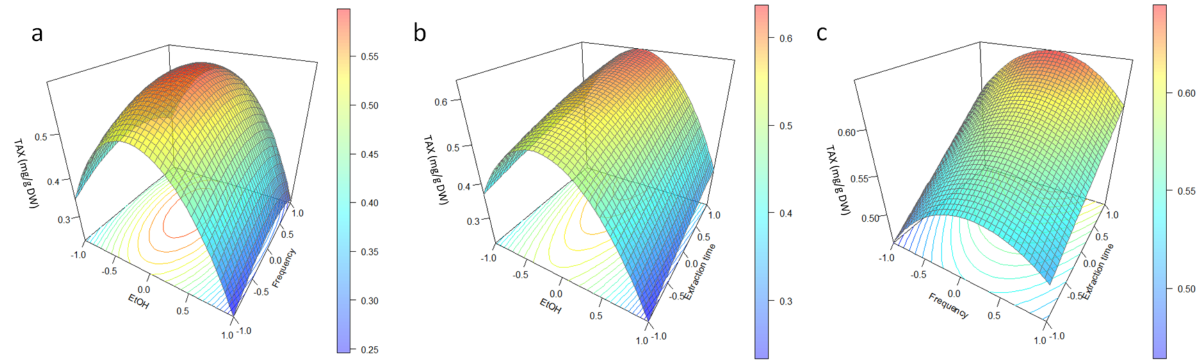

Figure S2 3D plots from the model predicted TAX extracted quantities from mature fruits of *Silybum marianum* as a function of (a) ethanol concentration and ultrasound frequency, (b) ethanol concentration and extraction duration, and (c) ultrasound frequency and extraction duration.

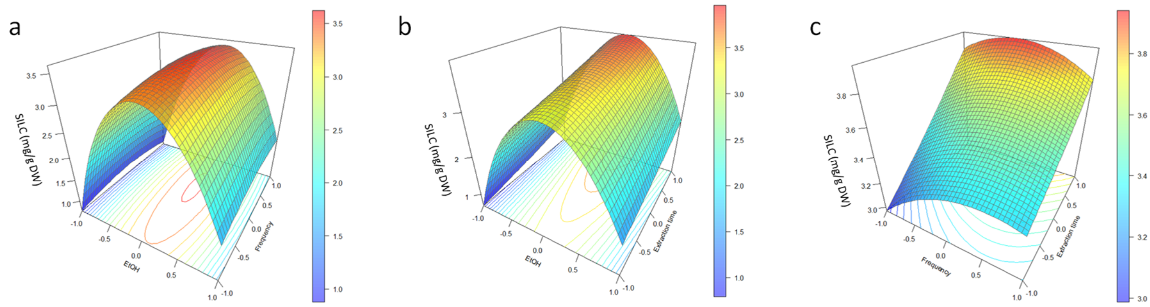

Figure S3 3D plots from the model predicted SILC extracted quantities from mature fruits of *Silybum marianum* as a function of (a) ethanol concentration and ultrasound frequency, (b) ethanol concentration and extraction duration, and (c) ultrasound frequency and extraction duration.

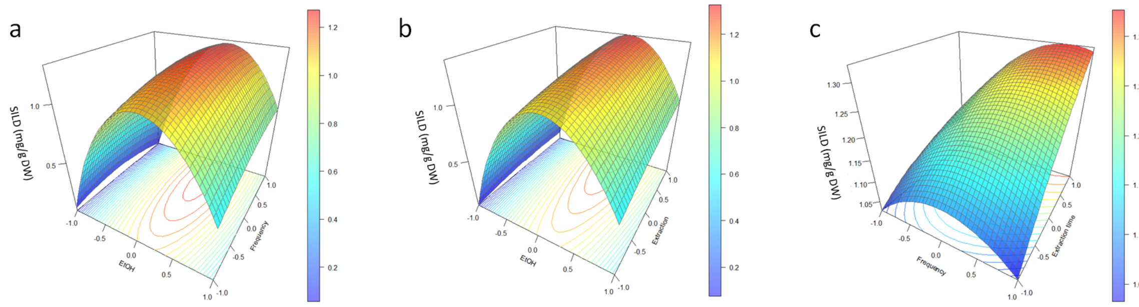

Figure S4 3D plots from the model predicted SILD extracted quantities from mature fruits of *Silybum marianum* as a function of (a) ethanol concentration and ultrasound frequency, (b) ethanol concentration and extraction duration, and (c) ultrasound frequency and extraction duration.

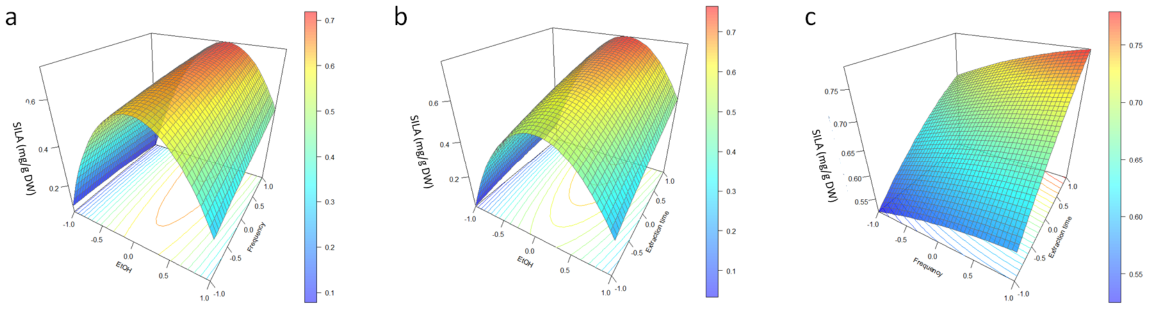

Figure S5 3D plots from the model predicted SILA extracted quantities from mature fruits of *Silybum marianum* as a function of (a) ethanol concentration and ultrasound frequency, (b) ethanol concentration and extraction duration, and (c) ultrasound frequency and extraction duration.

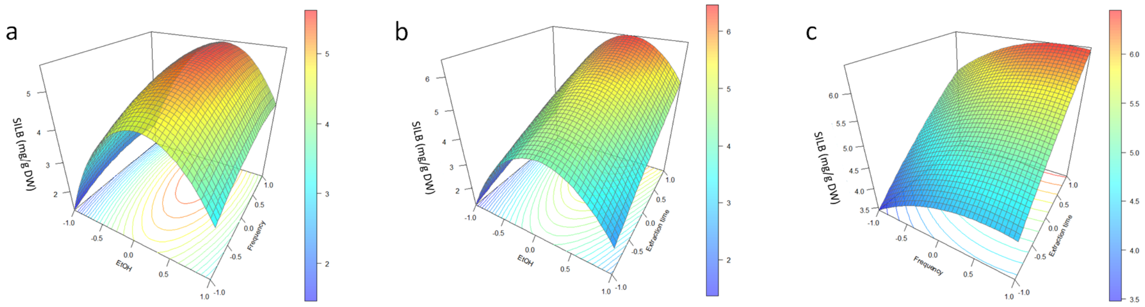

Figure S6 3D plots from the model predicted SILB extracted quantities from mature fruits of *Silybum marianum* as a function of (a) ethanol concentration and ultrasound frequency, (b) ethanol concentration and extraction duration, and (c) ultrasound frequency and extraction duration.

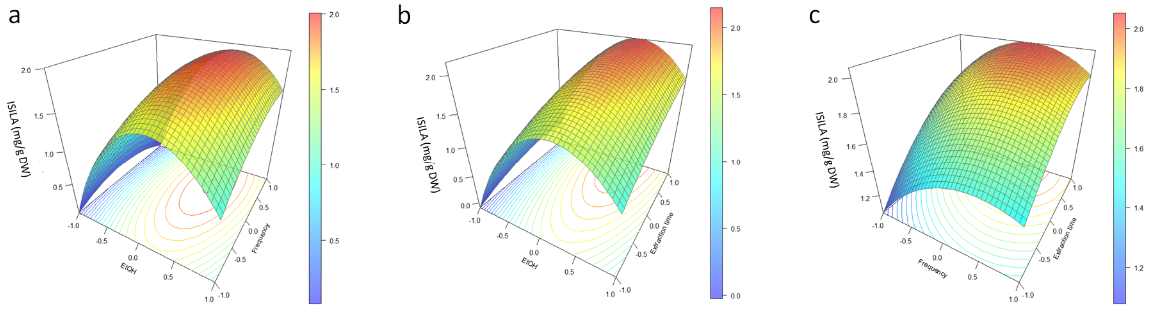

Figure S7 3D plots from the model predicted ISILA extracted quantities from mature fruits of *Silybum marianum* as a function of (a) ethanol concentration and ultrasound frequency, (b) ethanol concentration and extraction duration, and (c) ultrasound frequency and extraction duration.

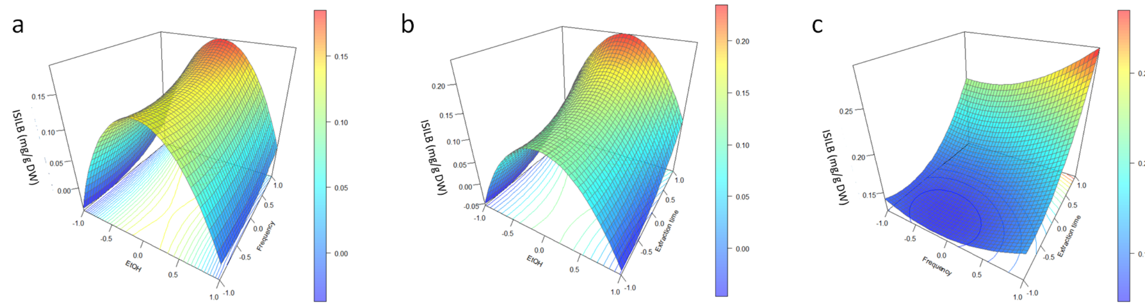

Figure S8 3D plots from the model predicted ISILB extracted quantities from mature fruits of *Silybum marianum* as a function of (a) ethanol concentration and ultrasound frequency, (b) ethanol concentration and extraction duration, and (c) ultrasound frequency and extraction duration.

Table S1 Results of full factorial design experiments for the extraction of TAX, SILC, SILD, SILA, SILB, ISILA and ISILB from mature fruits of *Silybum marianum*

Run ID	TAX	SILC	SILD	SILA	SILB	ISILA	ISILB
Run ID#1	0.24	0.06	0.46	0.19	1.29	nd	nd
Run ID#2	0.39	1.03	2.43	0.33	2.20	0.61	nd
Run ID#3	0.16	0.39	1.36	0.16	1.80	0.45	nd
Run ID#4	0.43	0.05	0.62	nd	1.46	nd	nd
Run ID#5	0.51	1.05	2.69	0.42	3.65	1.16	0.17
Run ID#6	0.21	0.59	1.59	0.30	2.66	1.16	nd
Run ID#7	0.40	nd	1.71	0.12	2.14	nd	nd
Run ID#8	0.42	0.74	2.97	0.44	2.79	1.05	0.04
Run ID#9	0.25	0.55	1.43	0.33	3.16	1.14	0.03
Run ID#10	0.46	0.05	1.07	0.07	1.57	nd	nd
Run ID#11	0.56	0.93	3.77	0.64	4.56	1.53	0.14
Run ID#12	0.25	0.42	1.51	0.27	2.87	0.81	nd
Run ID#13	0.30	0.04	0.40	0.01	1.06	nd	nd
Run ID#14	0.66	1.20	4.06	0.69	5.79	1.86	nd
Run ID#15	0.26	0.84	2.24	0.47	4.33	1.66	nd
Run ID#16	0.42	nd	nd	nd	2.07	nd	nd
Run ID#17	0.43	1.39	3.23	0.65	5.35	1.62	0.20
Run ID#18	0.26	0.73	1.75	0.49	4.08	1.79	0.04
Run ID#19	0.27	0.01	0.83	0.04	1.35	nd	nd
Run ID#20	0.64	1.31	4.21	0.85	6.59	2.14	0.25
Run ID#21	0.25	0.46	1.47	0.28	3.66	1.04	0.10
Run ID#22	0.54	nd	1.51	0.12	2.03	nd	nd
Run ID#23	0.65	1.24	4.06	0.75	7.27	2.43	0.31
Run ID#24	0.30	0.88	1.94	0.48	4.87	1.59	nd
Run ID#25	0.38	nd	1.06	nd	2.60	nd	nd
Run ID#26	0.68	1.52	4.14	1.09	7.52	2.49	0.55
Run ID#27	0.27	0.72	1.96	0.37	4.21	1.01	nd

Values are the means of 3 independent replicates expressed in mg/g DW. nd: not detected.

Table S2 Values, standard deviations and statistical analysis of the regression coefficients for the TAX, SILC, SILD, SILA, SILB, ISILA and ISILB extraction yield from mature fruits of *Silybum marianum* as a function of the 3 different variables (X1: ethanol concentration, X2: ultrasound frequency and X3: extraction duration).

**Source**	**TAX**	**SILC**	**SILD**	**SILA**	**SILB**	**ISILA**	**ISILB**
Constant	0.59***	1.23***	3.60***	0.67***	5.40***	1.89***	0.14*
X_1	-0.068**	0.30***	0.42*	0.14***	0.89***	0.59***	0.01^{ns}
X_2	0.015^{ns}	0.05^{ns}	0.06^{ns}	0.04^{ns}	0.45*	0.14^{ns}	0.02^{ns}
X_3	0.054*	0.09*	0.33*	0.09*	1.05***	0.28*	0.05^{ns}
X_1^2	-0.233***	-0.83***	-2.23***	-0.45***	-2.46***	-1.06***	-0.18*
X_2^2	-0.056^{ns}	-0.08^{ns}	-0.16^{ns}	-0.01^{ns}	-0.36^{ns}	-0.23^{ns}	0.02^{ns}
X_3^2	-0.011^{ns}	-0.03^{ns}	0.02^{ns}	-0.02^{ns}	-0.12^{ns}	-0.13^{ns}	0.04^{ns}
X_1X_2	-0.010^{ns}	0.07^{ns}	0.03^{ns}	0.05^{ns}	0.04^{ns}	0.14^{ns}	nd7^{ns}
X_1X_3	nd08^{ns}	0.05^{ns}	0.03^{ns}	0.04^{ns}	0.33^{ns}	0.07^{ns}	nd1^{ns}
X_2X_3	-nd8^{ns}	0.05^{ns}	-0.10^{ns}	0.01^{ns}	nd5^{ns}	-0.07^{ns}	nd1^{ns}

*** for p < nd1; ** for p < 0.01; * for p < 0.05; ns not significant.

Table S3 ANOVA results of the TAX, SILC, SILD, SILA, SILB, ISILA and ISILB extraction models

Source	TAX	SILC	SILD	SILA	SILB	ISILA	ISILB
Model F-value	8.03***	32.85***	14.73***	10.12***	8.48***	13.33***	2.70*
Lack of fit F-value	0.12 ns	0.027 ns	0.068 ns	0.087 ns	0.085 ns	0.062 ns	0.34 ns
R^2	0.810	0.946	0.886	0.843	0.852	0.876	0.589
adj R^2	0.709	0.917	0.826	0.759	0.774	0.810	0.371
CV %	0.415	0.342	0.118	0.342	0.322	0.270	0.895

R^2: determination coefficient; R^2 adj: adjusted R^2; CV variation coefficient value; *** significant p < nd1; ** significant p < 0.01; * significant p < 0.05; ns not significant.

Table S4 Individual antioxidant and antimicrobial activities vs RA contents in the 27 US extract samples.

Run ID#	CUPRAC[a]	AGE[b]	COLA[b]	ELA[b]
1	51.88	8.59	12.18	8.28
2	92.49	25.18	20.20	10.80
3	98.73	16.50	11.62	10.04
4	59.83	9.31	6.17	10.69
5	111.61	41.06	27.47	11.38
6	103.80	27.11	16.28	8.56
7	83.81	15.78	12.07	9.63
8	99.07	33.72	28.73	12.36
9	97.65	27.98	16.39	8.46
10	51.33	11.47	8.53	7.87
11	122.82	48.72	34.37	16.71
12	128.12	25.43	15.14	7.69
13	64.47	6.64	11.25	6.84
14	152.73	58.23	37.16	15.16
15	110.01	39.62	23.91	12.50
16	100.01	10.13	4.20	7.51
17	146.98	55.62	37.72	17.27
18	131.23	37.82	21.36	11.13
19	46.02	9.14	10.49	8.96
20	173.26	68.15	44.67	21.05
21	120.16	29.77	16.91	8.60
22	65.86	15.06	11.36	7.15
23	172.09	67.02	47.25	21.30
24	105.13	45.98	23.52	12.17
25	143.49	15.40	9.60	8.30
26	183.80	74.32	49.13	22.93
27	101.26	34.02	20.75	10.64

Values are means of 3 independent replicates; colours represent the relative activities or contents, from blue (for relative low activities or contents) to red (for relative high activities or contents); [1] extraction conditions are described in Table 2; Two antioxidant assays were conducted: CUPRAC (expressed as ascorbic acid equivalent antioxidant capacity (AEAC, in µM AEAC)) and the inhibition of advanced glycation end product (AGE) formation (expressed in % of inhibition relative to a control obtained by measuring the activity of the corresponding extraction solvent). Two anti-aging assays were conducted by determining the inhibition activity of each extracts toward collagenase (COL) and elastase (ELA) enzymes (expressed in % of inhibition relative to a control obtained by measuring the activity of the corresponding extraction solvent).
